# Antidepressant-Like Effect of *Geniposide* in Mice Exposed to a Chronic Mild Stress Involves the microRNA-298-5p-Mediated Nox1

**DOI:** 10.3389/fnmol.2020.00131

**Published:** 2021-02-03

**Authors:** Tianyu Zou, Jielin Zhang, Yongxiu Liu, Yiming Zhang, Kazuo Sugimoto, Cheng Mei

**Affiliations:** ^1^Department of Encephalopathy, Heilongjiang Academy of Chinese Medical Sciences, Harbin, China; ^2^Department of Dermatology, Heilongjiang Provincial Hospital Affiliated to Harbin Institute of Technology, Harbin, China; ^3^Department of Neurology, Dongzhimen Affiliated Hospital, Beijing University of Chinese Medicine, Beijing, China

**Keywords:** *geniposide*, depression, inflammation, mouse frontal cortex, mitochondria, reactive oxygen species, microRNA-298-5p, NADPH oxidase 1

## Abstract

Depression is a common mental disorder that presents a considerable challenge for public health. The natural product *geniposide* has neuroprotective effects on depression, but the underlying mechanism behind these effects had remained undefined. The present study was designed to investigate the role of microRNAs (miRs) in this mechanism. It studied mice with depression-like behavior established by exposure to chronic unpredictable mild stress (CUMS) for 2 months. The CUMS mice were intragastrically fed with *geniposide* at a dose of 10 ml/kg daily for two consecutive weeks. We monitored the depression-like behaviors of the CUMS mice by the forced swimming test (FST) and tail suspension test (TST). Then, we measured the cerebral expression of miR-298-5p and NADPH oxidase 1 (Nox1) mRNA in the CUMS mice by the RT-qPCR. The targeting relationship between miR-298-5p and Nox1 was evaluated by dual-luciferase reporter gene assay. The concentrations of adenosine triphosphate (ATP) and reactive oxygen species (ROS) were determined by the CellTiter-Glo^®^ and flow cytometry, respectively. The mitochondrial membrane potential (MMP) was detected using JC-1 staining. Moreover, the expression of inflammatory cytokines (TNF-α, IL-1β, IL-6, and TGF-β) was determined by ELISA, RT-qPCR, and western blot analysis. We found that miR-298-5p was poorly-expressed while Nox1 was highly-expressed in the brain tissues of the CUMS-induced mice. Intriguingly, *Geniposide* treatment reversed the behavioral abnormalities of CUMS mice, including shortened immobility time. *Geniposide* inhibited the Nox1 expression by increasing miR-298-5p levels. There were increased ATP content and MMP and reduced contents of ROS and inflammatory cytokines in the CUMS mice receiving *geniposide* treatment. Hence, this study revealed an antidepressant effect of *geniposide* on CUMS-induced depression-like behavior in mice by down-regulating the miR-298-5p-targeted Nox1. This highlights a novel candidate target for the treatment of depression.

## Introduction

Depression is a heterogeneous psychiatric disorder that poses challenges for clinical treatment due to its systemic complexity and lack of clear therapeutic targets (Wittenborn et al., 2016). Worse still, comorbid depression has been reported to enhance the morbidity rate of stroke, cardiovascular diseases, diabetes, and obesity (Penninx et al., 2013). With the dramatic acceleration in the pace of life in modern society, stress has become one of the most common environmental factors that cause depression (Athira et al., 2019). To date, the optimal choice in the treatment of depression is antidepressants (Kok and Reynolds, 2017). Unfortunately, not all patients benefit from available therapies, and others experience only short-term improvement. Thus, there are great challenges for depression therapeutics (Cuijpers, 2018), meaning studies need to identify novel and more effective therapeutic approaches.

*Geniposide* is a major bioactive extract from the fruit of *Gardenia jasminoides* that possesses antibacterial and anti-inflammatory properties (Wei et al., 2018). A previous study has illustrated the antidepressant effects of *geniposide* on chronic unpredictable mild stress (CUMS) in a rat model with depression-like behavior (Cai et al., 2015). A further study has demonstrated that *geniposide* treatment relieved depression-like behavior in a mouse model provoked by repeated restraint stress (RRS; Zhao et al., 2018). The differential regulation of microRNAs (miRs) expression has also been reported during stress. Thus, miRs might be promising targets of antidepressant treatments (Dwivedi, 2016). For instance, a previous study detected the dysregulation of miR-132 in patients with depression, which was linked to the loss of gray matter volume in the fronto-limbic network and cognitive impairments (Qi et al., 2018). Moreover, Wang et al. (2018) confirmed miR-155 as a significant factor in the development of depression and a novel target for antidepressant treatments.

Nox1 is a non-phagocytic form of 2, 4-dienoyl-CoA reductase 1 (NADPH) oxidase that plays a crucial role in depression-like behaviors (Ibi et al., 2017). The down-regulation of miR-25 significantly elevates the expression of Nox4 to mediate myocardial dysfunction (Varga et al., 2013). Genipin, the aglycone of *geniposide*, mediates the apoptosis of hepatoma carcinoma cells through NADPH oxidase-dependent generation of reactive oxygen species (ROS; Kim et al., 2005). However, the involvement and downstream therapeutic mechanism of *geniposide* in depression remained uncharted. Therefore, we designed this study to explore the correlation between *geniposide*, miR-298-5p, and Nox1 in depression.

## Materials and Methods

### Ethics Statement

The current study was performed with approval from the ethics committee in Heilongjiang Academy of Chinese Medical Sciences. All animal experiments were performed by following the Guide for the Care and Use of Laboratory Animals published by the US National Institutes of Health.

### Animal Model Establishment and Drug Administration

In total, 110 specific pathogen-free (SPF) C57BL/6J mice (male, aged 6–8 weeks, and weighing 20–30 g) were purchased from the Vital River Laboratory Animal Technology Co., Limited (Beijing, China). All mice were raised in the SPF animal laboratory with a relative humidity of 60–65% at a temperature of 22–25°C. Of these 110 mice, 10 were selected as normal controls, 10 normal mice were treated with *geniposide*, and the remaining 90 mice were used to establish the mouse model with depression-like behaviors by CUMS based on the previously described method (Leng et al., 2018; Sun et al., 2019) with minor modifications. The mouse models with depression-like behavior were exposed to the following stimulus cycle for 2 months: 4 h of body restraint, 24 h of food deprivation, 16 h of water deprivation, 4 h of cold water swim, 12 h of overnight illumination, 12 h of wet padding, and 24 h of the day/night inversion. The normal mice were kept under the same feeding conditions but did not receive any such stressors. The model with depression-like behavior was successfully established in 46 mice, with a success rate of 51%. Among these, 40 CUMS mice were selected for subsequent experiments.

Among these 40 CUMS mice, 10 CUMS mice were treated with *geniposide*, while 10 CUMS mice received no further treatment. Briefly, *geniposide* was first mixed with 0.5% sodium carboxymethylcellulose (CMC-NA) and fed to the mice intragastrically at a dose of 10 ml/kg daily for 2 consecutive weeks.

The remaining 20 CUMS mice received intravitreal injection with Nox1 knockdown (KD-Nox1) and the corresponding negative control (NC) vector using a modified AAV2 adenovirus (Ad; Hanbio, Shanghai, China). For this treatment, animals were intravitreally injected with 2μl conventional AAV2 vectors at a dose of 2 × 10^9^ total vg [diluted to a titer of 1 × 10^12^ vg/ml with serum-free, antibiotic-free Dulbecco’s modified Eagle’s medium (DMEM)] (Wassmer et al., 2017). The mice were then anesthetized by intraperitoneal injection of 0.05 mg/g pentobarbital sodium (P3761, Sigma-Aldrich, St. Louis, MO, USA). We then conducted surgical removal of the orbits, and the collection of 0.8–1.5 ml heart blood using an anticoagulated blood collection tube (367947, BD bioscience, New York, NY, USA). The mice were then euthanatized by cervical dislocation, followed by the collection of frontal cortex tissues.

### Behavioral Tests

Behavioral tests included the forced swimming test (FST) and tail suspension test (TST). In the FST, mice were placed in a glass cylinder with a diameter of 30 cm and a height of 25 cm. The depth of water was 15 cm and the water temperature was kept at 24°C. Then mice were forced to swim for 6 min. The TST was carried out under dark conditions in a colored Perspex box. The tail of mice was fixed at about 1 cm from the body on a crossbar elevated 35 cm above the ground. The head of mice was turned downward for 6 min followed by 1 min of free movement, during which the immobility time was recorded.

### Bioinformatics Analysis

miR and mRNA sequencing was conducted on the brain tissues of three untreated CUMS mice and *geniposide*-treated CUMS mice and three normal mice and CUMS mice. After quality control of the original data, the known miRs were identified by the BLAST program. The differential analysis was performed with the “DESeq” package in the R-language. Meanwhile, the known mRNAs were identified by the BLAST program. Then, the difference was analyzed by the “DESeq2” package in R-language. The downstream target gene of miR-298-5p was predicted by the TargetScan database^1^ and the miRWalk database^2^. The predicted results were then intersected using the Venn tool^3^ to identify the significantly up-regulated mRNAs in the brain of mice with depression-like behavior.

### Culture and Treatment of Frontal Cortex Neuron

Frontal cortex samples of normal and CUMS mice were incubated in 0.25% trypsin-ethylene diamine tetraacetic acid (EDTA; Sigma-Aldrich, St. Louis, MO, USA) at 37°C for 10 min. Then the tissues were incubated in a DMEM (Gibco, Thermo Fisher Scientific, MA, USA) containing 10% fetal bovine serum (FBS, ExCell Bio, Genetimes, Shanghai, China) for 5 min. The supernatant was discarded after centrifugation at 800× *g* for 5 min. Afterward, the isolated neurons were resuspended in a neurobasal medium (Gibco BRL, Grand Island, NY, USA) containing 1% FBS and 1× B27 supplement (NB/B27) followed by culture in a culture bottle coated with polylysine. Subsequently, the neurons from normal mice and CUMS mice were infected with the adenovirus (Ad) containing Nox1 overexpression (Ad-Nox1), short hairpin RNA (shRNA) against Nox1 (Ad-sh-Nox1), miR-298-5p inhibitor, miR-298-5p mimic, and their corresponding NCs (including Ad-NC, NC-inhibitor, and NC-mimic).

### Dual-Luciferase Reporter Gene Assay

The synthetic Nox1 3’untranslated region (UTR) gene fragment was introduced into the pGL3-reporter (Promega, Madison, WI, USA) using the endonuclease sites XhoI and BamH I. Then, the complementary sequence mutation site of the seed sequence was designed based on the Nox1 wild type (WT). The digested sequence was inserted into the pGL3-reporter vector using T4 DNA ligase. The constructed luciferase reporter plasmid Nox1 WT or mutant (MUT) was co-transfected into HEK293T cells with miR-298-5p mimic or NC-mimic, respectively. The cells were harvested and lysed 48 h after transfection. Luciferase activity of cells was determined using the Luminometer TD-20/20 detector (E5311, Promega, Madison, WI, USA) on the Dual Luciferase^®^ Reporter Assay System (E1910, Promega, Madison, WI, USA).

### RNA and Protein Analysis

For the detection of the miR and mRNA expression, total RNA was extracted from tissues and cells using the TRIzol (Invitrogen, Carlsbad, CA, USA). Then the extracted RNA was reversely transcribed into complementary DNA (cDNA) according to the instructions of the First Strand cDNA Synthesis Kit (Takara, Tokyo, Japan). Reverse transcription-quantitative polymerase chain reaction (RT-qPCR) was performed using a SYBR Premix Ex Taq kit (Takara, Tokyo, Japan) on an ABI Prism 7500 Fast Real-Time PCR system (Applied Biosystems Inc., Carlsbad, CA, USA). The expression of miR and mRNA was calculated according to the 2^−ΔΔCt^ method with glyceraldehyde-3-phosphate dehydrogenase (GAPDH; for mRNA) and U6 (for miR) used as internal references. The primer sequences are presented in Table 1.

**Table 1:** Primer sequence of RT-qPCR.

Target	Primer sequence
IL-1β	F: 5′-GCACACCCACCCTGCA-3′
	R: 5′-ACCGCTTTTCCATCTTCTTCTT-3′
IL-6	F: 5′-TCCAGAAACCGCTATGAAGTTC-3′
	R: 5′-CACCAGCATCAGTCCCAAGA-3′
TNF-α	F: 5′-CTCCAGGCGGTGCCTATG-3′
	R: 5′-GGGCCATAGAACTGATGAGAGG-3’
TGF-β1	F: 5′-GCGTATCAGTGGGGGTCA-3′
	R: 5′-GTCAGACATTCGGGAAGCAG-3’
miR-298-5p	F: 5′-CCCTTCTTGTCGGGAGGAGACGG-3′
	R: 5′-GTGCAGGGTCCGAGGT-3′
Nox1	F: 5′-CGCTCCCAGCAGAAGGTCGTGATTACCAAG-3′
	R: 5′-GGAGTGACCCCAATCCCTGCCCCAACCA-3′
GAPDH	F: 5′-TGCCCCCATGTTTGTGATG-3′
	R: 5′-TGTGGTCATGAGCCCTTCC-3′
U6	F: 5′-CTCGCTTCGGCAGCACA-3′
	R: 5′-AACGCTTCACGAATTTGCGT-35′

*Note: IL-1β, interleukin 1β; IL-6, interleukin 6; TNF-α, tumor necrosis factor α; TGF-β1; transforming growth factor β1; miR-298-5p, microRNA-298-5p; Nox1, NADPH oxidase 1; GAPDH, glyceraldehyde-3-phosphate dehydrogenase; RT-qPCR, reverse transcription quantitative polymerase chain reaction; F, forward; R, reverse*.

For detection of the protein expression, total protein was first extracted from tissues and cells using the radioimmunoprecipitation assay buffer (R0010, Solarbio, Beijing, China). The protein concentration was determined using a bicinchoninic acid protein concentration assay kit (P0011, Beyotime, Shanghai, China). Proteins were then separated by polyacrylamide gel electrophoresis and transferred onto 0.2-μm polyvinylidene fluoride membranes (ISEQ10100, Millipore, MA, USA). Tris-buffered saline containing 0.1% Tween-20 solution (D8340, Soleil, Beijing, China) and 5% skim milk powder was used to block the membranes. The membranes were then probed with the primary antibodies at 4°C overnight. Then, the membranes were re-probed with horseradish peroxidase (HRP)-labeled secondary goat anti-rabbit immunoglobulin G antibody (1:5,000, A0208, Beyotime, Shanghai, China) for 1 h at room temperature. The membranes were visualized in an enhanced chemiluminescence (ECL) system (C-DiGit^®^ Blot Scanner, Li-Cor, NE, USA) and analyzed using the Image J software. The primary antibodies from Proteintech (Rosemont, IL, USA) included anti-nuclear factor-kappa B (NF-κB; 1:1,000, 14220-1-AP), the internal control GAPDH (1:4,000, 10494-1-AP), and primary antibodies from Cell Signaling Technology (Danvers, MA, USA) were anti-phosphorylated (p)-extracellular signal-regulated kinase 1/2 (ERK1/2; 1:1,000, #4376), ERK1/2 (1:1,000, #9102), phosphorylated NF-κB (1:1,000, #4810), phosphorylated c-Jun NH2-terminal kinase (JNK; 1:1,000, #4668), JNK (1:1,000, #9252), phosphorylated mothers against DPP family member 2/3 (SMAD2/3; 1:1,000, #8828) and SMAD2/3 (1:1,000, #868).

### Enzyme-Linked Immunosorbent Assay (ELISA)

After completion of the behavioral test on the mice in each group, serum samples were harvested, placed in an anticoagulant tube, and allowed to stand at room temperature for 1 h. The samples were then centrifuged at 1,000× *g* for 15 min at 4°C to collect the supernatant. Tumor necrosis factor-α (TNF-α), interleukin (IL)-1β, and IL-6 ELISA kits (H052, H002, and H007, Nanjing Jiancheng Bioengineering Institute, Nanjing, Jiangsu, China) were then used to measure the levels of these cytokines. In brief, 50 mM carbonate coating buffer (pH 9.6) was used to dissolve the antigen (10–20 μg/ml), which was then added into a 96-well enzyme label plate (100 μl/well) for overnight incubation at 4°C. Then, 150 μl of 1% bovine serum albumin was added to each well for 1 h of blocking at 37°C, and 100 μl of serum was added into each well to incubate for 2 h at 37°C. Subsequently, the serum was incubated with 100 μl of diluted HRP-labeled secondary antibodies at 37°C for 1 h. Finally, the absorbance value of A405 was read on a microplate reader (BioTek ELx800, Biotek Winooski, VT, USA).

### Adenosine-Triphosphate (ATP) Content Determination

Cells were seeded in a 96-well plate at a density of 1 × 10^4^ cells per well. After cells were processed according to the transfection requirements, the medium of each well was changed to obtain a 100 μl volume in each well. The CellTiter-Glo^®^ Luminescent Cell Viability Assay kit (G7570, Promega, Madison, WI, USA) was used to prepare a working solution according to the manufacturer’s instructions. Cells were then lysed for 2 min by the addition of 100 μl CellTiter-Glo^®^ working solution to each well in the dark. Then, a 200 μl mixed solution was transferred to an opaque 96-well plate, with 200 μl mixed solution containing medium and working solution at a ratio of 1:1 used as a control well. The determination of concentration was performed on a 96-well fluorescence detector (GloMax^TM^, Promega, Madison, WI, USA).

### Mitochondrial Membrane Potential (MMP) Detection

The cells in each group were planted in a special Confocal culture dish (NEST Biotechnology Co., Wuxi, China) at a density of 1 × 10^4^ cells per dish. Then, the cells were treated by following the transfection requirements. According to the manuals of the JC-1 MMP assay kit, the culture solution was aspirated, and the cells were washed once with phosphate buffer saline. Then, 1 ml of cell culture medium was added to the cells. After that, a 0.1 ml JC-1 staining solution was added to each dish and mixed followed by incubation at 37°C for 20 min. During the incubation, a JC-1 staining buffer (1×) was prepared using 4 ml distilled water per 1 ml JC-1 staining buffer (5 ×) and pre-cooled in an ice bath. After the incubation at 37°C, the supernatant was discarded, and cells were washed twice with JC-1 staining buffer (1×). Lastly, 1 ml cell culture solution containing serum and phenol red was added to the cells followed by observation under a laser confocal scanning microscope (SP8, Leica, Wetzlar, Germany). When JC-1 monomers were determined, the excitation light was set to 490 nm and the emission light was set to 530 nm. When JC-1 polymers were determined, the excitation light was set to 525 nm and the emission light was set to 590 nm. When the JC-1 monomers were observed under fluorescence microscopy, we used the settings for green fluorescent protein (GFP) or fluorescein isothiocyanate (FITC). When JC-1 polymers were observed, we used settings of other red fluorescent markers, such as propidium iodide or Cy3. The presence of green fluorescence indicated a decrease in MMP and that cells were likely to be in the early stages of apoptosis. Red fluorescence indicated normal MMP and normal cell status.

### Reactive Oxygen Species (ROS) Detection

The cells in each group were seeded in a 96-well plate at a density of 1 × 10^4^ cells per well. An active oxygen detection kit (S0033, Beyotime, Shanghai, China) was used for ROS detection. Dichloro-dihydro-fluorescein diacetate (DCFH-DA) was diluted with a multi experiment matrix (MEM) at a ratio of 1:1,000 to a final concentration of 10 M. Cells were collected and suspended in diluted DCFH-DA to a concentration of 1 × 10^6^ cells/ml and incubated for 20 min at 37°C. Cells and probes were mixed and inverted every 3–5 min to ensure full contact. Cells were washed three times with MEM to remove residual DCFH-DA that had not entered cells. Detection was conducted at the final excitation wavelength of 488 nm and the emission wavelength of 525 nm using an LSR II flow cytometer (BD, NY, USA).

### Statistical Analysis

Data were analyzed using the Statistical Product and Service Solutions (SPSS) 21.0 software (IBM Corp. Armonk, NY, USA). The measurement data were expressed as the mean ± standard deviation. Data between the two groups conforming to normal distribution were evaluated by an unpaired *t*-test, and data with skewed distribution were evaluated by Mann-Whitney U. Data among multiple groups were compared using one-way analysis of variance (ANOVA) followed by Tukey’s *post hoc* test. Data among multiple groups in skew distribution were tested by the nonparametric Kruskal-Walis H test. The differences were deemed to be statistically significant at *p* < 0.05 and *indicated *p* < 0.05. The effect of size and power were calculated using the G*power software.

## Results

### *Geniposide* Was Capable of Alleviating Depression in CUMS Mice

To investigate the therapeutic effect of *geniposide* on depression, we evaluated the depression-like behaviors of CUMS mice, including immobility time in FST and TST. As presented in Table 2, compared with normal mice, the immobility time in FST and TST was significantly prolonged in CUMS-induced mice (in TST, effect size = 0.67, power = 0.81, and *p* < 0.001; in FST, effect size = 0.69, power = 0.81, and *p* < 0.001). In comparison to CUMS-induced mice, the immobility time in FST and TST was significantly decreased in CUMS-induced mice after *geniposide* treatment (in TST, effect size = 0.69, power = 0.88, and *p* < 0.001; in FST, effect size = 0.87, power = 0.88, and *p* < 0.001). Moreover, the immobility time in FST and TST did not exhibit any significant change in normal mice treated with *geniposide* in contrast to normal mice (in TST, effect size = 0.23, power = 0.42, and *p* > 0.05; in FST, effect size = 0.15, power = 0.37, and *p* > 0.05). Therefore, *geniposide* could alleviate the behavioral signs of the CUMS depression model.

**Table 2:** Effects of *geniposide* on chronic unpredictable mild stress (CUMS)-induced behavioral abnormalities.

Group	TST (s)	FST (s)
Normal (*n* = 10)	41.25 ± 4.11	75.85 ± 8.18
CUMS (*n* = 10)	143.25 ± 13.34***	187.73 ± 19.67***
Normal + *Geniposide* (*n* = 10)	43.58 ± 4.09	72.14 ± 7.85
CUMS *+ Geniposide* (*n* = 10)	97.63 ± 10.34^###^	136.51 ± 19.12^###^

Note: ***p < 0.001 vs. normal mice, ^###^*p* < 0.001 vs. CUMS mice. Data comparison was analyzed using one-way ANOVA. In TST, *F*_(3,36)_ = 298.6, *p* < 0.001; in FST, *F*_(3,36)_ = 137.5, *p* < 0.001.

### miR-298-5p Inhibited the Expression of Nox1 in Mouse Frontal Cortical Neurons

**Figure 1:**
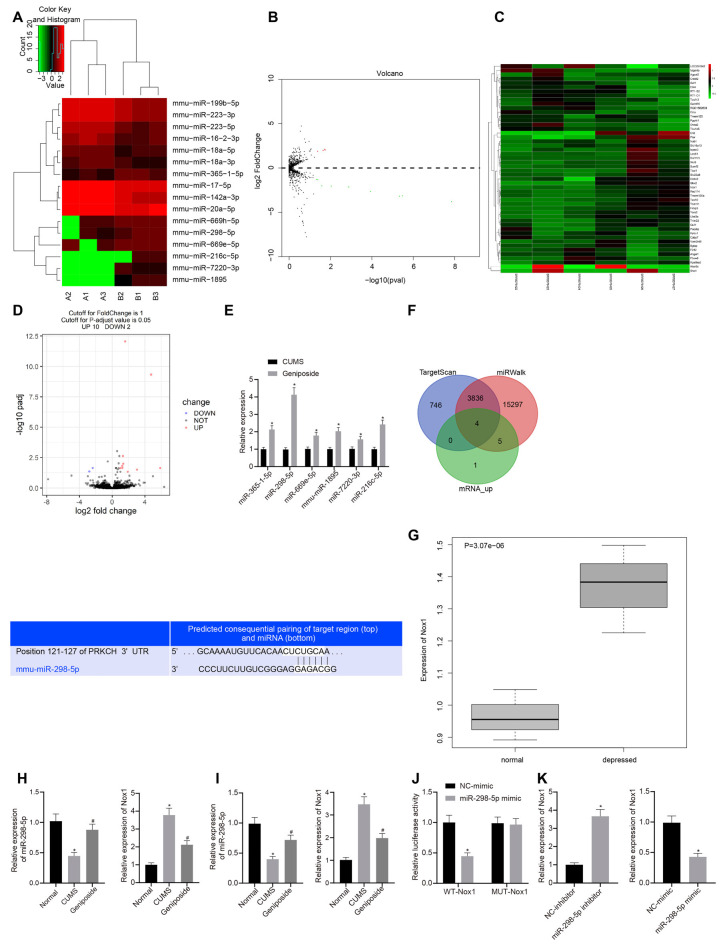
Nox1 was inhibited by miR-298-5p in mouse frontal cortical neurons. **(A)** The heat map of microRNAs (miRNAs) with significantly differential expression between chronic unpredictable mild stress (CUMS) mice and normal mice. **(B)** A volcano map of miRNAs with significantly differential expression between CUMS mice and normal mice. **(C)** The heat map of mRNAs with significantly differential expression between CUMS mice and normal mice. **(D)** A volcano map of mRNAs with significant differential expression between CUMS mice and normal mice. **(E)** Expression of miR-365-1-5p, miR-298-5p, miR-669e-5p, mmu-miR-1895, miR-7220-3p and miR-216c-5p in CUMS mice after *geniposide* treatment. **(F)** The target genes of miR-298-5p predicted by upregulated mRNAs in RNA sequencing data of the RAID and starBase databases, and the potential binding sites between miR-298-5p and NADPH oxidase 1 (Nox1). **(G)** High expression of Nox1 in the frontal cortex of CUMS mice with depression-like behavior. **(H)** miR-298-5p expression and Nox1 mRNA expression in mouse frontal cortex tissues determined by RT-qPCR (*n* = 10). The variance analysis of miR-298-5p expression: *F*_(2,98)_ = 103.9, *p* < 0.001; the variance analysis of Nox1 expression: *F*_(2,87)_ = 285.6, *p* < 0.001. **(I)** The expression of miR-298-5p and Nox1 mRNA in mouse frontal cortex neurons determined by RT-qPCR (*n* = 10). The variance analysis of miR-298-5p expression: *F*_(2,167)_ = 134.1, *p* < 0.001; the variance analysis of Nox1 expression: *F*_(2,20)_ = 284.4, *p* < 0.001. **(J)** The targeting relationship between miR-298-5p and Nox1 verified by dual-luciferase reporter gene assay. **(K)** The Nox1 mRNA expression in mouse frontal cortex neurons after overexpression or inhibition of miR-298-5p measured by RT-qPCR. *p < 0.05 vs. normal, NC-mimic, or NC-inhibitor. Data were all measurement data and expressed as mean ± standard derivation. ^#^*p* < 0.05 vs. CUMS. Comparisons between two groups were analyzed using unpaired *t*-test, and comparisons among multiple groups were analyzed using one-way ANOVA. The experiment was repeated three times independently.

To further explore the mechanism by which *geniposide* relieved depression-like behavior, we conducted a bioinformatics analysis. Accordingly, the brain tissues of three CUMS mice and *geniposide*-treated CUMS mice and those of three normal mice were subjected to miR and mRNA sequencing. After the original data were quality-controlled, the known miRs were identified by the BLAST. Then differential analysis was performed using the “DESeq” package in R language. We found multiple miRs (Figures 1A,B) and mRNAs (Figures 1C,D) with significantly differential expression between CUMS and normal mice. The downstream target genes of miR-298-5p were further predicted using the TargetScan and miRWalk databases. Then, we intersected the predicted results and the significantly up-regulated mRNAs in brain tissues of CUMS mice. RT-qPCR was used to verify the miR among the six identified miRNAs (miR-365-1-5p, miR-298-5p, miR-669e-5p, mmu-miR-1895, miR-7220-3p, and miR-216c-5p) associated with the *geniposide* treatment. Intriguingly, our results showed that miR-298-5p was significantly up-regulated in the brain tissues of CUMS mice treated with *geniposide* (Figure 1E, *p* < 0.01). We then predicted the downstream target genes of miR-298-5p using the TargetScan and miRWalk databases (Figure 1F). The intersection analysis was conducted on the predicted results and the up-regulated mRNAs. Our results revealed four intersected genes, among which Nox1 exhibited remarkably high expression in the brain tissues of CUMS mice (Figure 1G).

To verify the predicted results by the bioinformatics analysis, we measured the expression of miR-298-5p and Nox1 in the frontal cortex tissues using the RT-qPCR. Our results showed that, in contrast to the normal mice, the expression of miR-298-5p was down-regulated (*p* < 0.05), and the expression of Nox1 mRNA was up-regulated in the CUMS mice (*p* < 0.05; Figure 1H). Similar results were observed in primary mouse frontal cortex neurons (Figure 1I). After *geniposide* treatment, miR-298-5p expression was increased whereas the expression of Nox1 mRNA was decreased in CUMS mice (*p* < 0.05). On the other hand, the relationship between miR-298-5p and Nox1 was further confirmed by the dual-luciferase reporter gene assay (Figure 1J). In HEK293T cells, the luciferase activity of the WT 3’UTR of Nox1 was decreased upon treatment with miR-298-5p mimic (*p* < 0.05), while the luciferase activity in MUT 3’UTR of Nox1 showed no significant difference (*p* > 0.05). This suggested that miR-298-5p could directly target Nox1. Additionally, the primary mouse frontal cortex neurons were transfected with miR-298-5p-inhibitor or miR-298-5p-mimic. The Nox1 mRNA expression was elevated in neurons transfected with miR-298-5p-inhibitor but was reduced in neurons transfected with miR-298-5p-mimic (Figure 1K, *p* < 0.05). Collectively, the above-mentioned results confirmed the results of bioinformatics analysis that miR-298-5p could inhibit the Nox1 expression in mouse frontal cortex neurons.

### *Geniposide* Exerted Neuroprotective Effects by Increasing miR-298-5p and Decreasing Nox1 Expression

**Figure 2:**
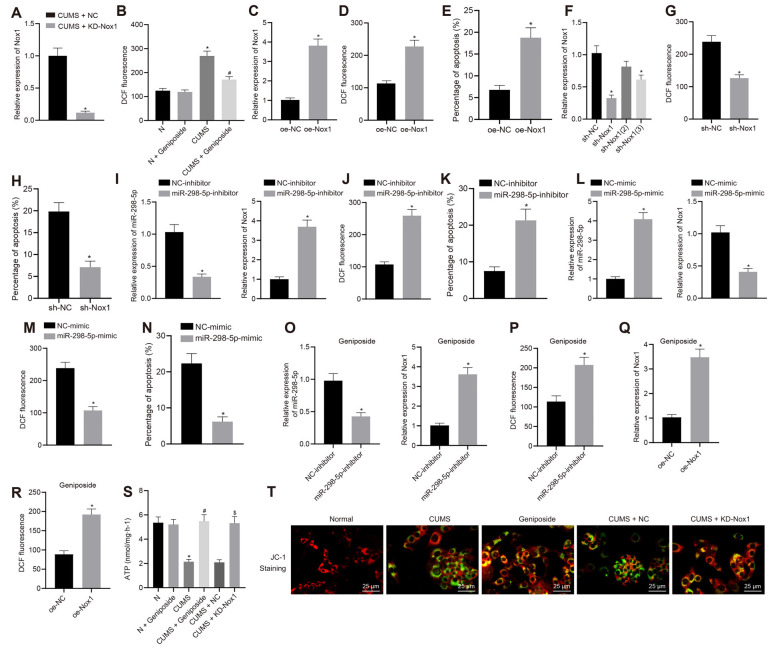
*Geniposide* reduced reactive oxygen species (ROS) content, inhibited oxidative stress and protected mitochondrial function in neurons by reducing Nox1 expression. **(A)** The Nox1 mRNA expression in the mouse frontal cortex determined by RT-qPCR (*n* = 10). *p < 0.05 vs. CUMS mice treated with NC. **(B)** The ROS content in the frontal cortex neurons of normal mice, CUMS mice, and *geniposide*-treated CUMS mice (*n* = 10). The variance analysis: *F*_(3,36)_ = 274.1, *p* < 0.001. *p < 0.05 vs. normal mice. ^#^*p* < 0.05 vs. CUMS mice. **(C)** The Nox1 mRNA expression of neurons transfected with oe-Nox1 or oe-NC determined by RT-qPCR. **(D)** The ROS content of neurons transfected with oe-Nox1 or oe-NC. **(E)** The apoptosis level of neurons transfected with oe-Nox1 or oe-NC detected by flow cytometry. *p < 0.05 vs. neurons transfected with oe-NC. **(F)** The Nox1 mRNA expression in neurons transfected with sh-Nox1 or sh-NC determined by RT-qPCR and the transfection efficiency of different shRNAs. The variance analysis: *F*_(3,35)_ = 39.68, *p* < 0.001. *p < 0.05 vs. neurons transfected with oe-NC. **(G)** The content of ROS in neurons transfected with sh-Nox1 or sh-NC. **(H)** The apoptosis level of neurons transfected with sh-Nox1 or sh-NC determined by flow cytometry. *p < 0.05 vs. neurons transfected with oe-NC. **(I)** miR-298-5p expression and Nox1 mRNA expression in neurons transfected with miR-298-5p-inhibitor or NC-inhibitor measured by RT-qPCR. **(J)** The ROS content in neurons transfected with miR-298-5p-inhibitor or NC-inhibitor. **(K)** The apoptosis level of the neurons transfected with miR-298-5p-inhibitor or NC-inhibitors detected by flow cytometry. *p < 0.05 vs. neurons transfected with NC-inhibitor. **(L)** miR-298-5p expression and Nox1 mRNA expression in neurons transfected with miR-298-5p-mimic or NC-mimic measured by RT-qPCR. **(M)** The ROS content of the neurons transfected with miR-298-5p-mimic or NC-mimic detected by flow cytometry. **(N)** The apoptosis level of the neurons transfected with miR-298-5p-mimic or NC-mimic detected by flow cytometry. *p < 0.05 vs. neurons transfected with NC-mimic. **(O)** miR-298-5p expression and Nox1 mRNA expression in neurons from *geniposide*-treated CUMS mice after transfection with miR-298-5p-inhibitor or NC-inhibitor determined by RT-qPCR. **(P)** The ROS content in the neurons from *geniposide*-treated CUMS mice after transfection after transfection with miR-298-5p-inhibitor or NC-inhibitor. *p < 0.05 vs. neurons from *geniposide*-treated CUMS mice transfected with NC-inhibitor. **(Q)** The Nox1 mRNA expression in neurons from *geniposide*-treated CUMS mice after transfection with oe-Nox1 or oe-NC determined by RT-qPCR. **(R)** The ROS content in neurons from *geniposide*-treated CUMS mice after transfection with oe-Nox1 or oe-NC detected by flow cytometry. *p < 0.05 vs. neurons from *geniposide*-treated CUMS mice transfected with oe-NC. **(S)** The ATP content in the neurons of different treatments. The variance analysis: *F*_(5,12)_ = 47.55, *p* < 0.001. **(T)** MMP in the neurons with different treatments (×400). *p < 0.05 vs. neurons from normal mice. ^#^*p* < 0.05 vs. neurons from CUMS mice. ^$^*p* < 0.05 vs. neurons from CUMS mice treated with NC. Data were measurement data and expressed as the mean ± standard deviation. The unpaired *t*-test was used to analyze comparisons between two groups, and comparisons among multiple groups were analyzed by one-way ANOVA, followed by Tukey’s *post hoc* test. The experiment was repeated three times independently.

To further explore how *geniposide* worked through control of Nox1 expression, we knocked down Nox1 in CUMS mice. Assays of mRNA expression of Nox1 in the mouse frontal cortex revealed that the Nox1 mRNA expression in CUMS mice was decreased after KD-Nox1 treatment compared to CUMS mice (Figure 2A). Our results from the behavioral function tests revealed that the immobility time in TST and FST was reduced in CUMS mice with Nox1 knockdown (Table 3; in TST, effect size = 10.36, power = 1, and *p* < 0.001; in FST, effect size = 6.30, power = 0.98, and *p* < 0.001).

**Table 3:** Effects of Nox1 knockdown on the behavioral functions of CUMS mice.

Group	TST (s)	FST (s)
CUMS + NC (*n* = 10)	159.47 ± 12.98	165.34 ± 21.22
CUMS + KD-Nox1 (*n* = 10)	54.34 ± 6.13***	62.38 ± 9.16***

Note: ***p < 0.001 vs. CUMS mice treated with sh-NC. Data comparison was analyzed by *t*-test.

The results of flow cytometry analysis showed that the ROS level in the mouse frontal cortex neurons of CUMS mice was higher than that in normal mice (*p* < 0.05), which was blocked by *geniposide* treatment (*p* < 0.05; Figure 2B). Compared to the CUMS mice, CUMS mice treated with *geniposide* exhibited reduced ROS levels in the frontal cortex neurons (*p* < 0.05), while normal mice treated with *geniposide* exhibited no significant difference in ROS level in comparison to the normal mice (*p* > 0.05). Additionally, mouse frontal cortex neurons from normal mice were transfected with oe-Nox1 and sh-Nox1. The results revealed increased Nox1 mRNA expression (Figure 2C) and ROS levels (Figure 2D) following transfection with oe-Nox1, accompanied by increased cell apoptosis (Figure 2E, Supplementary Figure 1A; *p* < 0.05). Moreover, in neurons of normal mice with Nox1 knockdown, the transfection efficiency of different sh-RNAs [sh-Nox1, sh-Nox1 (2), sh-Nox1 (3)] was first evaluated. Compared with the neurons transfected with sh-NC, Nox1 mRNA expression was decreased in neurons of mice transfected with sh-Nox1 (reduced 68.32%) and sh-Nox1 (3) (reduced 40.16%; Figure 2F, *p* < 0.05). The sh-Nox1 with the highest transfection efficiency was selected for subsequent experiments. Further experiments indicated that, in neurons isolated from CUMS mice, the sh-Nox1 treatment resulted in decreased ROS (Figure 2G, *p* < 0.05) and decreased apoptosis levels (Figure 2H, Supplementary Figure 1B; *p* < 0.05). Neurons were then transfected with miR-298-5p-inhibitor or miR-298-5p-mimic to evaluate the role of miR-298-5p in *geniposide* treatment. In neurons from normal mice, transfection with miR-298-5p-inhibitor reduced the miR-298-5p expression (Figure 2I) but increased the Nox1 mRNA expression (Figure 2I), ROS level (Figure 2J), and cell apoptosis (Figure 2K, Supplementary Figure 1C; *p* < 0.05). However, in neurons from CUMS mice, the miR-298-5p expression (Figure 2L) was significantly increased, but the Nox1 mRNA expression (Figure 2L), ROS level (Figure 2M), and cell apoptosis were decreased (Figure 2N, Supplementary Figure 1D; *p* < 0.05) after transfection with miR-298-5p-mimic. Additionally, the rescue experiments further validated that the low expression of miR-298-5p reversed the effect of *geniposide* in neurons from CUMS mice: transfection with miR-298-5p-inhibitor diminished miR-298-5p expression (Figure 2O), elevated Nox1 mRNA expression (Figure 2O), and ROS level (Figure 2P; *p* < 0.05) in neurons. Moreover, in the neurons isolated from *geniposide*-treated CUMS mice, the overexpression of Nox1 reversed the inhibitory effect of *geniposide* on the expression of Nox1 mRNA (Figure 2Q) and the ROS level (Figure 2R; *p* < 0.05). Therefore, we speculated that *geniposide* can reduce the expression of Nox1 by increasing the expression of miR-298-5p in the mouse frontal cortex neurons, thereby reducing the ROS content and protecting neurons.

The ATP content and MMP were detected to reflect the energy metabolism and early apoptosis of neurons. Compared with the neurons from normal mice, the ATP content of neurons from CUMS mice was decreased (Figure 2S), while MMP was also observed to be reduced (green fluorescence increased; Figure 2T, *p* < 0.05). *Geniposide* increased the ATP content (Figure 2S) and MMP (green fluorescence decreased; Figure 2T, *p* < 0.05) in neurons from CUMS mice. On the contrary, ATP content showed no remarkable changes in the *geniposide*-treated normal mice when compared to normal mice (Figure 2S; *p* > 0.05). Meanwhile, ATP content (Figure 2S) and MMP were increased (green fluorescence reduced; Figure 2T) by knocking down the Nox1 in neurons from CUMS mice (*p* < 0.05). Therefore, *geniposide* can reduce the ROS content, inhibit oxidative stress, and protect the mitochondrial function of neurons by reducing the Nox1 expression.

### *Geniposide* Reduced Oxidative Stress and Inflammation in CUMS Mice

**Figure 3:**
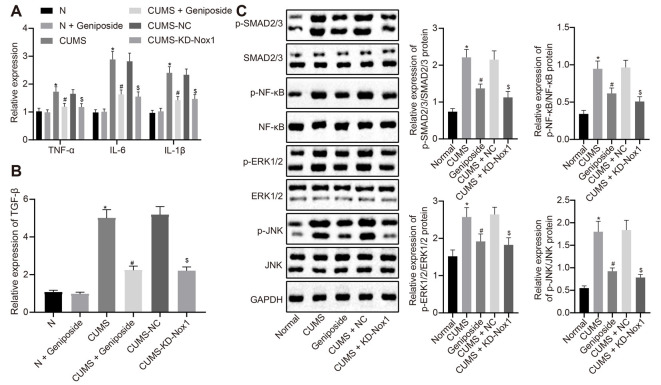
*Geniposide* reduced oxidative stress in CUMS mice by reducing ROS production and inflammation. **(A)** The levels of TNF-α, IL-1β and IL-6 mRNA in frontal cortex tissues after different treatment determined by RT-qPCR (*n* = 10). The variance analysis of TNF-α level: *F*_(5,54)_ = 58.46, *p* < 0.001. The variance analysis of IL-1β level: *F*_(5,54)_ = 153.00, *p* < 0.001. The variance analysis of IL-6 content: *F*_(5,54)_ = 173.00, *p* < 0.001. **(B)** The TGF-β mRNA level in the frontal cortex tissues after different treatment determined by RT-qPCR (*n* = 10). **(C)** The expression of SMAD2/3, NF-κB, JNK and ERK1/2 and their corresponding phosphorylation in the frontal cortex tissues after different treatment (*n* = 10). The variance analyses of phosphorylated SMAD2/3/SMAD2/3 protein: *F*_(4,160)_ = 44.18, *p* < 0.001, phosphorylated NF-κB/NF-κB protein: *F*_(4,88)_ = 35.99, *p* < 0.001, phosphorylated ERK1/2/ERK1/2 protein: *F*_(4,65)_ = 58.39, *p* < 0.001, and phosphorylated JNK1/2/JNK1/2 protein: *F*_(4,142)_ = 158.2, *p* < 0.001. *p < 0.05 vs. normal mice. ^#^*p* < 0.05 vs. CUMS mice. ^$^*p* < 0.05 vs. CUMS mice treated with NC. Data were all measurement data and expressed as mean ± standard deviation. Data among multiple groups were analyzed by one-way ANOVA, followed by Tukey’s *post hoc* test, and the experiment was repeated three times independently.

Compared with the normal mice, the serum levels of the inflammatory factors IL-1β, IL-6, and TNF-α of CUMS mice were increased (*p* < 0.001), but this increase was abolished after *geniposide* administration to the CUMS mice (For TNF-α serum level, effect size = 10.46, power = 0.98, and *p* < 0.001; for IL-1β serum level, effect size = 8.69, power = 0.89, and *p* < 0.001; for IL-6 serum level, effect size = 11.15, power = 1, and *p* < 0.001). Additionally, there was no marked difference in the serum levels of IL-1β, IL-6, and TNF-α between the normal mice and *geniposide*-treated normal mice (*p* > 0.05). The serum levels of IL-1β, IL-6, and TNF-α were decreased in CUMS mice after treatment with KD-Nox1 (Table 4; For TNF-α serum level, effect size = 12.49, power = 1, and *p* < 0.001; for IL-1β serum level, effect size = 8.32, power = 1, and *p* < 0.001; for IL-6 serum level, effect size = 11.69, power = 1, and *p* < 0.001). Besides, the mRNA expression of IL-1β, IL-6, TNF-α, and TGF-β was increased in the frontal cortex tissues of CUMS mice in contrast to that in normal mice while it was reduced in CUMS mice following *geniposide* treatment (*p* < 0.05). Furthermore, no significant difference was found in the mRNA expression of IL-1β, IL-6, TNF-α, and TGF-β between the normal mice and *geniposide*-treated normal mice (*p* > 0.05). In CUMS mice, Nox1 knockdown led to a reduction in the mRNA levels of IL-1β, IL-6, TNF-α, and TGF-β in the frontal cortex tissues (Figures 3A,B; *p* < 0.05).

**Table 4:** The serum levels of IL-1β, IL-6 and TNF-α in mice.

Group	TNF-α (pg/ml)	IL-1β (pg/ml)	IL-6 (pg/ml)
Normal (*n* = 10)	109.73 ± 9.28	64.23 ± 8.16	49.19 ± 5.38
CUMS (*n* = 10)	337.81 ± 32.78***	215.13 ± 15.39***	178.16 ± 18.69***
Normal + *geniposide* (*n* = 10)	114.67 ± 11.68	60.59 ± 9.35	55.63 ± 6.89
CUMS *+ geniposide* (*n* = 10)	144.11 ± 10.49^###^	72.05 ± 6.33^###^	54.35 ± 5.65^###^
CUMS + NC (*n* = 10)	379.88 ± 31.71	193.09 ± 14.22	182.34 ± 17.38
CUMS + KD-Nox1 (*n* = 10)	96.22 ± 5.16^$$$^	48.93 ± 5.13^$$$^	32.19 ± 4.44^$$$^

Note: ***p < 0.001 vs. normal mice, ^###^*p* < 0.001 vs. CUMS mice, ^$$$^*p* < 0.001 vs. the CUMS mice treated with sh-NC. Data comparison was analyzed by one-way ANOVA. The variance analysis of TNF-α content: *F*_(5,54)_ = 396.6, *p* < 0.001. The variance analysis of IL-1β content: *F*_(5,54)_ = 503.3, *p* < 0.001. The variance analysis of IL-6 content: *F*_(5,54)_ = 365.4, *p* < 0.001.

Western blot analysis illustrated an increase in the expression of phosphorylated SMAD2/3, phosphorylated NF-κB, phosphorylated ERK1/2, and phosphorylated JNK in the frontal cortex tissues of CUMS mice compared with the normal mice (Figure 3B, *p* < 0.05), which was blocked after CUMS mice were treated with *geniposide* (Figure 3C, *p* < 0.05). In CUMS mice, the expression of phosphorylated SMAD2/3, phosphorylated NF-κB, phosphorylated ERK1/2, and phosphorylated JNK was down-regulated in the frontal cortex tissues following knockdown of Nox1 (Figure 3C, *p* < 0.05). Therefore, we anticipated that *geniposide* can alleviate the activation of inflammatory factors and related pathways associated with the depression model. In summary, *geniposide* improved the depression-like behavior in CUMS mice by inhibiting ROS production, rescuing mitochondrial damage, and attenuating inflammatory responses through reducing Nox1 expression.

## Discussion

Depression is a highly prevalent psychiatric illness that limits psychosocial activity and critically affects the quality of life. In 2008, depression was ranked as the third major cause of disease burden worldwide by the World Health Organization (Malhi and Mann, 2018). Accumulating evidence has suggested an association between depression and inflammation (Reddy et al., 2018; Zwicker et al., 2018). Interestingly, *geniposide*, an iridoid glycoside, has been indicated to possess several biological activities, including anti-inflammation and antidepressant effects (Wang et al., 2016; Zhang et al., 2017a). However, the potential of *geniposide* for treating depression remains requires further research. The objective of the present study was to investigate the mechanism by which *geniposide* could repress depression. Our data suggested that *geniposide* could potentially alleviate the depression-like behaviors in mice by decreasing the Nox1 through enhancing the expression of miR-298-5p.

Initially, our results showed that *geniposide* reversed impaired and depression-like behavior in the CUMS-induced mouse model, as suggested by a shortened immobility time in TST and FST. Accordingly, a previously reported study has demonstrated that after CUMS, numerous impairments in mood, cognition, and memory, and decreases in hippocampal neurogenesis were observed. There was also cortical and limbic brain region atrophy, excessive activation of the noradrenergic system, increases in hippocampal inflammatory proteins, and hypothalamic-pituitary-adrenal axis disturbances (Antoniuk et al., 2019). Due to its close resemblance to the behavioral characteristics of patients with depression and related emotional disorders, CUMS is the most commonly applied and reliable method for a rodent model of depression (Araujo et al., 2018).

On the other hand, *geniposide* treatment has been illustrated to alleviate the depression-like behaviors of rats induced by CUMS by regulating the hypothalamus-pituitary-adrenal axis (Cai et al., 2015). Although it is confirmed that *geniposide* has therapeutic effects in the treatment of depression, the underlying mechanism of this therapeutic action remains unclear and several studies have attempted to unravel its regulatory mechanisms. For instance, there are indications that *geniposide* may be an agonist of the glucagon-like peptide-1 receptor (GLP-1R), thus exerting an anti-depressive effect on CUMS mice (Gong et al., 2014; Sun et al., 2018). Moreover, another study found that *geniposide* repressed neuronal apoptosis and reversed the depression-like behavior induced by repeated restraint stress in mice by regulating the GLP-1R/AKT signaling pathway (Zhao et al., 2018). Furthermore, it was also observed that *geniposide* increased the BDNF expression to attenuate depression-like behavior and cellular alterations in the hippocampus of streptozotocin-treated mice (Wang et al., 2016). Collectively, the above-described findings suggest that *geniposide* may possess an antidepressant role through a variety of mechanisms in the CUMS-induced mouse model with depression-like behaviors, and therefore merit further study.

To further investigate the effects of *geniposide* on depression, we treated CUMS mice with *geniposide*. Intriguingly, our results manifested a significant decrease of the contents of IL-1β, IL-6, TNF-α, and TGF-β, and expression of phosphorylated SMAD2/3, phosphorylated NF-κB, phosphorylated ERK1/2, and phosphorylated JNK in frontal cortex tissues. Consistently, the dysfunction of inflammatory cytokines has been implicated in the pathogenesis and treatment of depression (Dallé et al., 2017; Köhler et al., 2017). Furthermore, the levels of IL-1β, IL-6, TNF-α, and TGF-β are positively correlated with the symptoms of depression (Oliveira Miranda et al., 2014; Shariat et al., 2017). Additionally, the down-regulation of phosphorylated SMAD2/3, phosphorylated NF-κB, phosphorylated ERK1/2, and phosphorylated JNK also proved to be crucial indicators of the improvement of depression symptoms (Dow et al., 2005; Su et al., 2017; Zhang et al., 2017b). Hence, the above findings validate the antidepressant effect of *geniposide*.

We found that miR-298-5p was poorly expressed in CUMS mice while Nox1 exhibited a high expression. We further observed that miR-298-5p could potentially inhibit the expression of Nox1. Confirming our results, another recent study has also identified that cerebrospinal fluid concentrations of miRs such as miR-16 were distinctly lower in rats with depression-like behaviors than in normal rats (Song et al., 2015). Other research has also shown the down-regulation of miR-101b in the prefrontal cortex tissues of rats with depression-like behaviors (Wei et al., 2016). Noxs have been confirmed to be promising targets for combating neurodegenerative disorders (Ma et al., 2017; Tarafdar and Pula, 2018). Nox1 has been reported to be up-regulated in the ventral tegmental area of the brain in association with depression-like behaviors (Ibi et al., 2017). In CUMS mice treated with sh-Nox1, the ROS level is consistently reduced but an elevated concentration of ATP content and MMP was observed. These results suggested that Nox1 knockdown had a similar effect on oxidative stress as *geniposide* treatment. ROS is considered to possess a crucial role in both the normal physiology and pathology of erobic organisms (Li et al., 2016). A previous study has demonstrated the increased ROS production in depression (Maes et al., 2019). Interestingly, ATP has been implicated in the astrocytic modulation of depression-like behavior, and its up-regulation exhibited antidepressant-like effects (Cao et al., 2013). ATP deficiency in relation to impairment of mitochondrial function is also implicated in depression (Karabatsiakis et al., 2014). As key indicators of mitochondrial function, MMPs exhibit decreased concentration in the hippocampus after induction of mitochondrial dysfunction, which results in depression-like behaviors (Sakamuru et al., 2016; Chen et al., 2017). In the present study, *geniposide* reduced the expression of Nox1 by elevating the expression of miR-298-5p. Thus our study manifested the antidepressant-like effects of *geniposide* in the CUMS model. Similarly, it has been documented that *geniposide* treatment can potentially elevate the expression of miR-145–5p to protect cells from inflammatory injuries (Ma et al., 2019). Moreover, Nox1 has been indicated as the non-phagocytic isoform of superoxide-producing NADPH oxidase, suggesting it as a potential source of ROS (Iwata et al., 2018). Kazumi Iwata et al. outline that the aglycone of *geniposide* like genipin modulated the generation of ROS, and regulated the apoptosis of hepatoma carcinoma cells *via* NADPH oxidase (Kim et al., 2005). Our study has reported that *geniposide* potentially decreases the expression of Nox1 by upregulating miR-298-5p and indicated an antidepressant-like action.

## Conclusion

**Figure 4:**
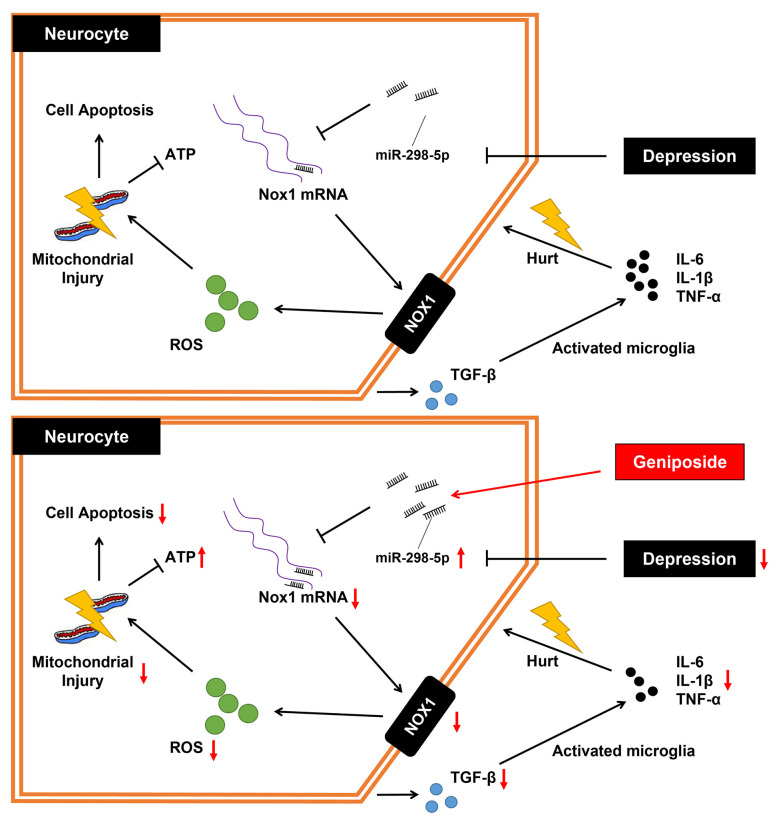
The mechanistic diagram of how *geniposide* regulated the miR-298-5p/Nox1 axis in alleviating depression-like behavior. Without *geniposide* treatment, mice with depression-like behavior might show more signs of inflammation. *Geniposide* treatment can alleviate depression in mice by increasing miR-298-5p expression but reducing Nox1 expression, ROS production, mitochondrial damage, and inflammatory responses.

In summary, the key findings from this study have supported the notion that *geniposide* treatment augmented the miR-298-5p expression and reduced Nox1 expression, which further elevated concentrations of ATP and MMP while decreasing the level of ROS and inflammation. Thus, it ultimately ameliorated the CUMS-induced depression-like behaviors in mice (Figure 4). Our study has suggested that *geniposide* holds the potential of a novel therapeutic candidate for depression management.

## Data Availability Statement

The raw data supporting the conclusions of this article will be made available by the authors, without undue reservation, to any qualified researcher.

## Ethics Statement

The animal study was reviewed and approved by the ethics commission of the Heilongjiang Academy of Chinese Medical Sciences.

## Author Contributions

TZ and JZ designed the study. YL, YZ and KS collated the data, carried out data analyses and produced the initial draft of the manuscript. CM contributed to drafting the manuscript. All authors have read and approved the final submitted manuscript.

## Conflict of Interest

The authors declare that the research was conducted in the absence of any commercial or financial relationships that could be construed as a potential conflict of interest.

## Footnotes

^1^http://www.targetscan.org/vert_71/

^2^http://mirwalk.umm.uni-heidelberg.de/

^3^http://bioinformatics.psb.ugent.be/webtools/Venn/
