# Supplementary figures and images for: Antidepressant-Like Effect of Geniposide in Mice Exposed to a Chronic Mild Stress Involves the microRNA-298-5p-Mediated Nox1
Source: Front Mol Neurosci. 2021 Feb 3;13:131. doi: 10.3389/fnmol.2020.00131 (PMC7886707; doi:10.3389/fnmol.2020.00131)

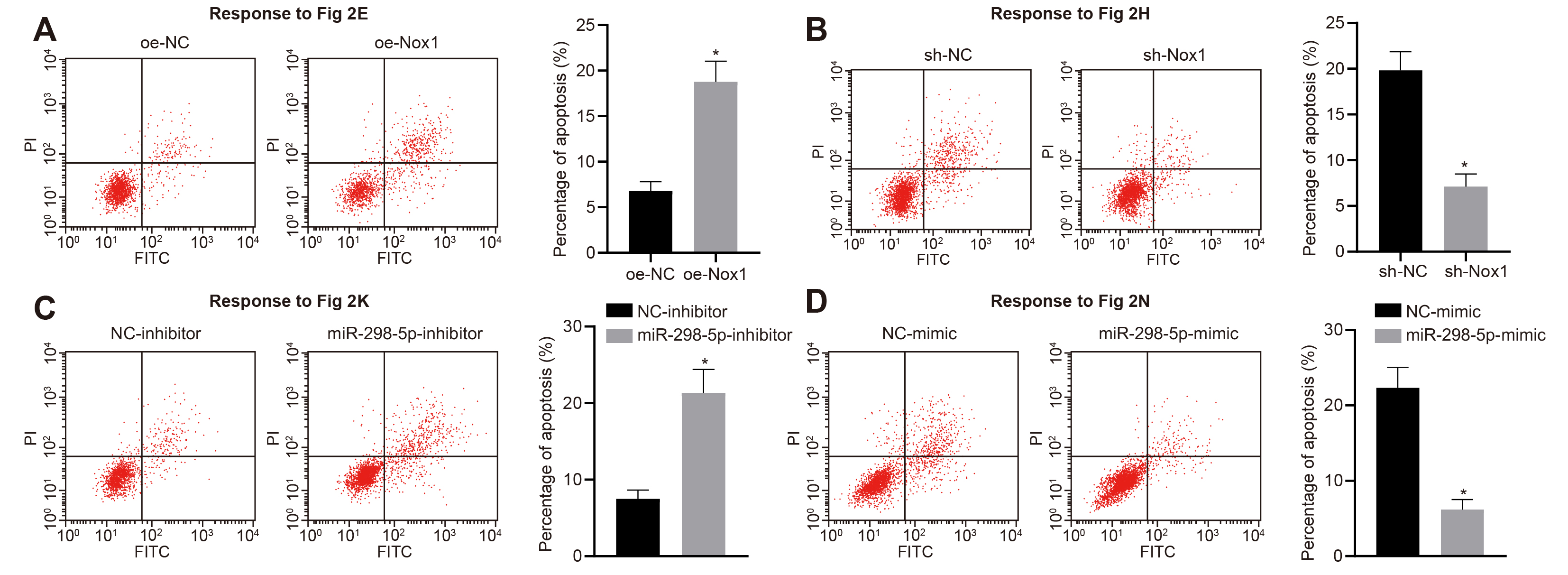

Supplement: Supplementary file 1 [file Image_1.JPEG]
